# Indents

**Published:** 1920-02

**Authors:** 


### INDENTS

**☞Brief Articles of Technical or Scientific Value will receive notice and comment. Contributions invited.**

NASAL BREATHING A PREVENTIVE OF CARIES. C. N. Peacock, in the *British Dental Journal* for August 15th, says that because of the more continuous flow of saliva over the teeth when patients breathe through the nose and not through the mouth the saliva has a better chance to cleanse the tooth surfaces by the flushing action and it also neutralizes acidity. The saliva also tends to saturate the enamel with lime salts as Bunting and Rickert suggest.

THE RADIOGRAPH SALESMAN. If there is at present no law prohibiting laymen from making medical and dental diagnoses, from radiographs alone, it is high time that such a law should be enacted. These men are really only selling radiographs, though it is a fact that they do append a diagnosis with each set of pictures. But this is only because that aids in the sale of the pictures. The person who purchases a photograph of himself is somewhat of a judge of its excellence. But since he can not "read" radiographs, forsooth they must be "read" for him. In this the patient is deceived. He applies for the service under the expectation that he will discover whether or not his teeth are diseased or, perhaps, may be the cause of his systemic troubles. However excellent the radiographs themselves may be, the accompanying diagnosis can not be anything but worthless, since the "reader" of the picture has no medical nor dental clinical experience.

A patient asked a dentist recently: "Why do you charge me twenty-five dollars for two radiographs, when I brought you ten radiographs of my mouth that cost me only ten dollars?" The reply was: "I do not sell radiographs, and I have no intention of delivering to you the radiographs that I have made. I am charging you for this consultation and I took the radiographs to aid me in coming to a conclusion. These radiographs, therefore, must remain in my own records, but they will be accessible to any physician or dentist that you may wish to consult at any time."—Dr. Ottoleregui, in November *Items of Interest.*

---

ACID-PRODUCING BACTERIA AND DENTAL CARIES. We are now justified, we believe, in concluding that conditions that facilitate the lodgment and growth of bacteria on any particular enamel surface, repeatedly or continuously, favor the action there of all the acid-producing types of oral bacteria. Of these bacteria, streptococci are usually, if not always, the most numerous at the beginning of the process, because of their normal abundance in the oral cavity. But as these different kinds of bacteria continue to grow at the given point of focalization on a tooth, B. acidophilus produces substances that favor its own growth, but interfere with the development and action of S. viridans, a fact that explains the shift in the proportions in these two types of organisms, as caries progress, which have heretofore puzzled and misled all who have studied this problem.

Our results indicate, further, that lactic acid would not be a suitable ingredient of a dentifrice, because it might favor the growth of the most destructive organisms involved in dental decay. Our findings suggest, also, that sour milk, with its relatively high content of lactic acid, to say nothing of its lactic acid-producing organisms, may be far more detrimental to the teeth than has hitherto been supposed.

These results indicate that important advances might be made in our knowledge of the cause, and in our means for the control of dental caries, if we could learn the nature of the acid or acids produced by S. viridans and other oral streptococci, and if we could determine the effects of these acids and their dental products in studies similar to the one we summarize.—W. J. Gies, in *Cosmos.*

A Lingual Matrix. Dr. G. S. Junkerman describes in *Dental Facts* for January a method of making the lingual surface of proximal cavities in the incisal teeth when the cavity has involved the lingual and incisal surfaces. Adapt a piece of thin steel to lingual contour extending it well over the margins of the cavity. Place an ivory separator between the teeth after applying the dam, then place the matrix in position and force it to place with softened modeling compound so the compound will engage in the separator and when set securely fix the matrix in place. The excess compound which forces its way into the cavity is then removed so as to clear the cavity, leaving a simple cavity with the lingual wall supplied by the smooth metal surface. A silicate or gold foil filling can be more easily made and the lingual surface will require little or no finishing. By extending the matrix over the incisal surface this surface can also be restored more easily. Some care will have to be taken to properly form and adopt the matrix as well as to back it up securely with the compound. The cavity margins will also need to be made free so the filling material can be closely adapted. The idea might be worked advantageously with distal cavities in the cuspids and lower teeth, including the bicuspids, where access can be had from the labial surfaces.

---

Importance of Oral Hygiene, by T. P. Hyatt, D.D.S. I should not be accused of a desire to exaggerate when I tell you there is no one subject to-day so important to the spiritual, the moral, the intellectual and the physical development of the race as this question of oral hygiene. I believe I state the truth when I say this.

Do you realize that life is dependent on three things—the air we breathe, the water we drink and the food we eat? Take away any one, or any two, or all these three elements, and the human being could not live. I should like you to fully appreciate the entire significance of that statement. The child needs air to live; the child needs liquid to drink; the child needs food to eat—and all of these pass through the mouth or are affected by the condition of the mouth before being taken into the digestive apparatus of the body to be digested and assimilated, to build up the growing body of the child. You know full well that vast sums of money are being spent each year in every town and city of every state of this union so that the water conveyed to the population shall be as pure as it is humanly possible to make it. You know vast sums are spent every year by our government to inspect the food that it may be as pure as it is humanly possible to have it, before it is conveyed to the child. You know every good mother and housekeeper will take care that water and food are served or prepared in clean utensils before they are given to the child and taken into the mouth—the mixing chamber for the food and liquid before it is taken into the digestive apparatus to be changed into those forms necessary to build up the different parts of the human body.

Of all the millions of dollars that have been expended for that purpose, how much has been spent upon the mouth of the child—the mixing chamber, the recipient of this food and water? I shall show you a picture of the waterworks of Greater New York. You will see the mighty work that has been done by man to convey pure water to the inhabitant. When you realize, as every conscientious dentist realizes, the appalling condition of the mouths of the children in Greater New York alone, and the physical impossibility of rectifying that condition by mere curative measures, you will realize that those men of my profession who have a little view into the future appreciate the tremendous importance of preventive measures, because we know that while it is not possible to absolutely prevent decay of the teeth, it is possible to reduce the number of decayed teeth by the teaching of oral hygiene, and that it will well repay every community and every city to take up this work in a conscientious and intelligent way.

Furthermore, we are learning—the medical profession as well as the dental profession—that the physical development of the child depends largely on the kind of food the child receives, just as this building depends upon the materials used in its construction. If they be good, the building will be strong; if they be poor, the building will be weak. Where the body is debilitated by improper nourishment, all its parts are rendered susceptible to disease—the teeth among them—so there is the relation of cause and effect. We know that through the lack of proper power to chew the food, there takes place a lack of development of the bony structure of the head. The average person does not realize the enormous amount of muscular power used in the mastication of food. The strongest muscles of our body are those that are used for the purpose of mastication, and the bones of the child are soft and pliable; and as these muscles are exercised in mastication, they pull on the bony structure of the skull and increase its size.

There have been experiments conducted with rabbits, where the teeth of one rabbit were destroyed on the right side, and the teeth of another rabbit destroyed on the left side, with a third rabbit left with the full denture as a control; and when the rabbits grew to full size they were killed and their skulls examined. It was found that the skull of the rabbit was not developed as well on the side where the teeth were destroyed as on the side where the teeth were left intact.—*Cosmos* for November.

---

"VINCENT'S ANGINA." The first step in treatment is to rid the mouth of all broken-down roots and infected teeth, if any, followed by a thorough scaling and polishing of the teeth, just as much being done at the first sitting as possible. No harm is done by removing sloughing gum tissue. The teeth are then isolated by packing the mouth with large wads of absorbent cotton. With a blunt silver probe, sodium perborate is worked around the teeth, well under the free margin of the gums, using the dry powder, the blood acting as a solvent for the powder. The powdered sodium perborate should be applied every day until a negative report comes from the laboratory. Then if the gums show improvement, the perborate should be discontinued, and tincture of iodin applied daily on the dry gums, packing the mouth with cotton as before. Patients are instructed to keep the mouth and teeth thoroughly clean, and to use peroxid of hydrogen mouthwash freely.

Following the first treatment the characteristic odor should disappear, the patients are relieved of pain, the gums begin to acquire a healthy appearance, and loosened teeth become firm. No disastrous effects have been observed from this treatment, and, without exception, patients are ready for discharge in from seven to ten days—just as soon as three successive negative reports are received and the mouth is well. Silver nitrate and chromic acid are not to be compared with the sodium perborate. When the occasion demands it, some stubborn patch may be treated with a little trichloracetic acid, but even this is used but little, the sodium perborate being satisfactory.—Dr. N. H. McDonald, Army Dental Surgeon in France, in *Dental Cosmos,* January, 1920.

---

Is Alcohol a Stimulant? Until comparatively recently, alcohol was regarded as a respiratory and cardiac stimulant. Whisky was probably the most popular domestic remedy for such occasional upsets as fainting, in which stimulation was presumably required. The popular idea that alcohol is a tone stimulant has so often proved to be untenable on the basis of scientific evidence that it seems almost superfluous to refute the mistaken notion anew. No one will deny that a feeling of relief often follows the use of alcohol in conditions in which stimulation seems to be indicated. In such cases, however, it has been shown to act merely as an irritant to the mucous membranes of the mouth and throat, promoting thus a beneficent local reaction before the alcohol has had time to be absorbed and stimulate in any way the depressed function.

There would be little occasion to refer again to the subject at this time had not a layman of prominence, Mr. G. E. Flint, recently, in a fiery volume, vigorously defended alcohol as a stimulant. Any tyro in physiology can find contradictions in Flint's book which, as a recent reviewer remarked in *The Journal*, "sometimes quotes confidently as fact what is absolutely not true." Consider, for example, this statement (page 155):

"That alcohol is very easily digested is proved by the fact that the carbohydrates can not be digested as such, but only after they have been changed into sugar and finally into alcohol."

To the author of such statements, seemingly endorsed by a medical expert, alcohol is distinctly a "heart stimulant." And then there is further instruction: "That alcohol is not a stimulant has never been proved."

In animal experimentation the use of anesthetics, which are commonly employed to insure painless effects in such studies, may interfere with the interpretation of the observations in which alcohol, itself a narcotic, is concerned. Lately, satisfactory methods of investigation without the use of the anesthetic when alcohol is being tested have been devised. Hyatt, who has given the most recent report, found that when alcohol is given by mouth there is a rapid rise in blood pressure, followed by an immediate return to normal. This is a purely local effect due to irritation of the gustatory nerves and swallowing movements. The same result can be obtained with dilute acids. When the alcohol was introduced directly into the circulation without preliminary contact with mucous membranes, there was either no effect or a fall in pressure. An anesthetic dose may actually be given by introduction in this manner into the circulation, to which it must find its way in any mode of administration, without any stimulation of the heart or respiration. Even when moderate doses of 40

per cent. alcohol are introduced into the stomach slowly by the use of a gastric sound, no change in pressure is produced.

These experiments confirm the earlier studies of the same sort which Brooks has described in *The Journal.* Why need the subject receive further argument? In refuting the claim that alcohol is in any sense a direct stimulant of the heart, a distinguished British committee reporting to the Central Control Board thus formulated the status of the controversy:

"The fact that the beneficial effect appears almost immediately and long before any significant amount of alcohol can have been absorbed and carried to the heart, is evidence for this local and indirect nature of the action. Its use in these circumstances is, therefore, comparable with that of smelling salts, or the irritating fumes of burnt feathers, traditionally employed for the same purpose. When, in conditions of more protracted weakness of the heart, the administration of alcohol has a beneficial effect, this must be attributed mainly to its mildly narcotic and sedative action, relieving the centers which modify the action of the heart from the disturbing influence of pain and anxiety. The promotion of a patient's comfort, the relief of mental strain, may be an essential element in the treatment of disease, and an important factor in recovery. It does not, however, justify the description of alcohol as a 'stimulant' of the heart."—Editorial in *Journal A. M. A.*, Dec. 27, 1919.

---

BRIDGEWORK AND THE MOBILITY OF TEETH. When we deal with a normal mouth and normal teeth the mobility of each individual tooth is quite apparent and beneficial.

However, when it comes to the replacing of lost teeth by either fixed or removable bridgework, conditions have changed. We will never be able to restore this lost normal condition in any case, whatever we may attempt to do, and what we may do is merely a "crutch."

In all cases where lost teeth are to be replaced, the first or vital question is not that of a fixed, removable or movable-removable bridge. *No!* It is always a question, what is *the need* for this particular case? The crowns, the fixed or removable bridges or plates are after all only the means to a satisfactory end.

There are innumerable cases where fixation of the remaining teeth by splint or fixed bridges are of the greatest need and importance, and again as many cases where fixation of the teeth would be detrimental to the abutments.—Dr. Hovestadt, in *Items of Interest.*

---

Devitalization of Pulp in Bridgework. The devitalization of teeth has been overdone in crown and bridgework in the past, not only for fixed, but also for removable bridgework. And in spite of all the claims of the advantages for all this removable bridgework, the fact still remains that more pulps are destroyed to-day by the men who practice removable bridgework, in order to accommodate the various attachments for these bridges, than is done by men who practice fixed bridgework. This is indeed a very vital point to consider. I believe we should do all we possibly can to prevent the destruction of vital pulps.—Dr. Hovestadt, in *Items of Interest.*

---

Saddle Bridges. The so-called clasp bridges should not come under bridgework at all. It is nothing new. This work was called clasp dentures and partial plates fifty and more years ago, and that is the place where it belongs to-day and should be so considered.

The argument in favor of clasp saddle bridges, also of large fixed or removable bridges *with saddles,* is not founded on sound principles. The value of saddles is misunderstood. The saddle does not play such an important part in the support of a bridge and it does not relieve the bridge abutments of the entire stress of the bite and mastication. In fact, some saddles are a disadvantage and often promote absorption and increase the stress on the remaining normal organs.

Take any clasp bridge and cut from the clasps the lugs which rest on the occlusal plane of the abutments or the clasped teeth. What will happen? No matter how well the saddle has been fitted this saddle bridge will sink at once and the occlusion and articulation of the bridge is lost either immediately or within a few days. Saddles on removable bridges are supported by abutments and when freed from the same, will sink and hasten absorption faster than when no saddle is present. Ask yourself, "What causes absorption under saddles?" and you have the answer to all this.—Dr. Hovestadt, in *Dental Items.*

---

SEPTIC BRIDGEWORK. As to septic dentistry, I want to say that a well-constructed fixed bridge is far less septic than most of the removable or movable-removable bridges, with their tubes, joints and box attachments to the teeth or roots.

This does not mean that I want to condemn removable bridgework. I simply point out to you some of the erroneous conceptions of this removable bridgework craze.

Dentists who have made bad fixed bridges in the past, will also make bad removable bridges. For years past I have devoted my *entire time* to this line of work. My practice is limited to bridgework. For twelve years I conducted college clinics and received bridge patients from all parts of the United States, from other dentists, and with the knowledge gained of fixed and removable bridgework, observing the results of my own and that of other men's work, from all states of the union, I am enabled to form this conclusion.

I must honestly confess that I do not see in the removable bridgework the remedy for all the ills and the shortcomings that have been laid at the door of fixed bridgework. We have to look further and correct many of our erroneous views on this subject to help the profession and the public.

It is neither the fixed nor the removable bridgework that we should push to the front, but rather we should use our energy to obtain a greater knowledge of classification, articulation and restoration of the lost normal conditions in individual cases.

Our greatest need to-day in crown and bridgework is a proper classification of bridges and partial dentures. As I have said before, whichever of these three we may use, it is only a means to a satisfactory end.

The suggestion to have bridgework done only by men who have taken a special post-graduate course would not solve the problem. What seems to be most needed *in training* is the classification of mouths with missing teeth and a more thorough study of tooth anatomy and articulation.—Dr. Hovestadt, in *Dental Items of Interest.*

---

FOCAL INFECTION AND THE EYES. According to a London daily paper, Mr. W. R. Ackland, L.D.S., M.R.C.S., Eng., was responsible for the following interesting announcement: "I know a woman who had eyes of different color, one grey and the other brown; but two months after a front tooth with an abscess beneath it had been extracted one eye was changed to the same color as the other." Assuming that the story is not a piece of journalistic embroidery, it would be of distinct value to get from Mr. Ackland himself or some other reliable observer an authentic account of the case. Inflammation caused by the abscess, it was said, affected the delicate nerves of the eye and caused discoloration of the pupil (iris ?), which had a brown and muddy appearance, although its natural color was grey.

Interviewed by the newspaper representative, the usual "eminent London specialist" said that people's eyes are occasionally of different colors, but he had never known the eyes of an adult to change color. Another doctor was reported to have said it was conceivable that an abscess beneath either of the "eye teeth" could interfere with the blood supply to one of the eyes, and so affect the color of the iris. The removal of the abscess might then be followed by a restoration of the eye to its normal color.—*Dental Record.*

---

THE DENTAL HYGIENIST. Thoughtful mothers are recognizing the need of dental service for the big boy and littlest girl, but what is the use of having a Little Fairy in your home if her teeth are improperly cared for and allowed to decay because of lack of proper care?

This is work that could be put into the hands of capable assistants, provided an oral hygiene law could be passed in all states licensing the dental hygienist. The dentist would then have more time for constructive and surgical work.

The greatest asset a dentist can have is a set of the famous "white pearls" in the mouth of every one of his patients. They are a walking advertisement for him. And no man can afford to overlook anything that will tend to spread his credit and increase his practice.

This is a day of specialization in everything, be it making bread pudding, politics or cleaning teeth. And the man who specializes in children's work has a big future before him. He has opened his box and, like the Lady Pandora, he is overwhelmed by what he got. But he needs time for self-development. The oral hygienist is bound to come; and in these days it is best to help push, instead of trailing the crowd. In time, perhaps, the government may even recognize the need of a "welfare measure."

Why not have a Forsythe or Rochester infirmary for the care of children's teeth in every large city? There are Eastmans and Forsythes everywhere ready and willing to interest themselves in this great work, if the dentist will do his part.

It's up to you, Mr. Dentist. Get out and pass this law. Let your local societies know just what you think of it. Don't sit and think what a good idea it is.—H. J. Bosworth, in *Dental Facts.*

